# Chemosensitivity of radioresistant cells in the multicellular spheroids of A549 lung adenocarcinoma

**DOI:** 10.1186/1756-9966-28-72

**Published:** 2009-06-02

**Authors:** Degang Shi, Genming Shi, Gang Huang, Jiren Zhang, Eric Lartigau

**Affiliations:** 1 Cancer Center, Zhujiang Hospital, Southern Medical University, Guangzhou 510282, PR China; 2 Département Universitaire de Radiothérapie, Centre Oscar Lambret, 59020 Lille Cedex, France; 3 Department of Chemotherapy, The First Affiliated Hospital, College of Medicine, Zhejiang University, Zhejiang, 310003, PR China; 4 Department of Nuclear Medicine, Renji Hospital, Shanghai Jiaotong University, Shanghai, 200001, PR China

## Abstract

**Background:**

The relapse of cancer after radiotherapy is a clinical knotty problem. Previous studies have demonstrated that the elevation of several factors is likely in some way to lead to the development of treatment tolerance, so it is necessary to further explore the problem of re-proliferated radioresistant cells to chemotherapeutic agents. In the present study, we aimed to investigate the chemosensitivity of radioresistant cells originated from the multicellular spheroids of A549 lung adenocarcinoma.

**Methods:**

After irradiated with 25 Gy of 6 MV X-ray to A549 multicellular spheroids, whose 10th re-proliferated generations were employed as radioresistant cells, and the control groups were A549 parental cells and MCF7/VCR resistant cells. The chemo-sensitivity test was made by six kinds of chemotherapeutic drugs which were DDP, VDS, 5-Fu, HCP, MMC and ADM respectively, while verapamil (VPL) was used as the reversal agent. Then the treatment effect was evaluated by MTT assay, and the multidrug resistant gene expressions of *mdr1 *and *MRP *were measured by RT-PCR.

**Results:**

Both A549 parental cells and A549 derived radioresistant cells were resistant to DDP, but sensitive to VDS, 5-Fu, HCP, MMC and ADM. The inhibitory rates of VPL to these two types of cell were 98% and 25% respectively (P < 0.001). In addition, without drugs added, the absorbance value (A value) of A549 parental cells was 2-folds higher than that of their radioresistant cells (P < 0.001). As to the MCF7/VCR cells, they were resistant to DDP and VDS, but slight sensitive to MMC, ADM, 5-Fu, and HCP with 80% of inhibitory rate to VPL. The subsequent RT-PCR demonstrated that the *Mdr1*/β2-MG and *MRP*/β2-MG of all A549 cells were about 0 and 0.7 respectively, and those of MCF7/VCR cells were 35 and 4.36.

**Conclusion:**

The chemosensitivity of A549 radioresistant cells had not changed markedly, and the decreased sensitivity to VPL could not be explained by the gene expression of *mdr1 *and *MRP*. It is possible that the changes in the cell membrane and decreased proliferate ability might be attributed to the resistance. Unlike multidrug resistance induced by chemotherapy, VPL may be not an ideal reverser to radioresistant cells. Therefore, the new biological strategy needs to be developed to treat recurring radioresistant tumor in combination with chemotherapy.

## Backgrounds

In patients with breast cancer, 4–47% may have local tumor relapse after chemotherapy and ionizing radiation therapy, this may be related to the sub-clinical focuses and resistant cell population, indicating bad prognosis [1]. Because the radiation dose may be lethal in case of relapse of the cancer after radiotherapy, a second time of radiation therapy would not be favored but chemotherapy would be an alternative in such cases. Therefore, it is necessary to explore the problem of re-proliferated radioresistant cells to chemotherapeutic agents [2].

Multicellular spheroid (MTS) is a three-dimensional structure formed by cancer cells, which could be used for radio-biological study and bioassay on drug sensitivity in vitro. The results obtained from this assay are closely mimic in vivo setting [3,4]. The microenvironment and cell cycle between A549 lung adenocarcinoma MTS and single layers are different [5]. Our former article had shown that the cell cycle retardation during G_2_-M phase became increased with increase of the irradiation dose, and only a few cells survived, proliferated and relapsed after prolonged subculture. The growth of radioresistant descendant cells was slow with low sensitivity to radiation [6]. Whether the change of drug sensitivity to chemotherapeutic agent in re-proliferated radioresistant cells may result in reduction and resistant, or sensitive, or the same as the primary cells is a problem worth to further investigate. In general, the mechanism of radioresistance and chemotherapy tolerance may have a common basis, and tumor cells at different cell cycle phase may have different degree of sensitivity to radiation and chemotherapeutic agents. For instance, cells in proliferate stage may be more sensitive. The survival of a few polyploidy giant cells in tumor after irradiation is perhaps due to p53 gene mutation resulting from DNA damage. The repairmen of tumor cells and tolerance to DNA damage form the basis of tolerance in the survived re-proliferated cells [7]. Radiation can also influence the apoptosis and some gene expression in regulating the cell cycle, e.g. C-Jun NH2-terminal kinase (JNK), protein kinase C (PKC), nerve ceramide cascade protein [8], survivin (an inhibition substance of membranous structure in the apoptosis protein family) [9] and CD40 activating signal [10], etc. The elevation of the above factors is likely in some way to lead to the development of tolerance.

In this study, MTS formed by A549 lung adenocarcinoma cells was used as the experimental model to assess chemosensitivity of radioresistant cells. A549 MTS was first treated with irradiation of 6 MV X-ray, then the susceptibility of radioresistant regrowth cells to chemotherapeutic agents and their multidrug resistance gene expression were analyzed thereafter.

## Methods

### Culture and irradiation of A549 MTS

6MV X-ray was used for single irradiation to A549 MTS, with irradiation dosage 15, 20, 25 and 30 Gy respectively and dosage rate 200 cGy/min. Then the MTS was cultured according to the conventional MTS culture methods [3,6], and the culture liquid was changed weekly. Living re-proliferated cells were noted 40 days after irradiation of 25 Gy or 30 Gy [6], with the radioresistant cells being the 10th generation cell after 25 Gy irradiation. Other irradiation groups were mainly 15 Gy ^32^P internal irradiation and 6MV X-ray external irradiation [11].

### Six kinds of chemotherapeutic drugs and verapamil in culture solution

The six kinds of chemotherapeutic agents were Cisdiaminodichloro-platinum (DDP), vindesin (VDS), 5-Fluorouracil (5-Fu), Hydroxycamptothecine (HCP), Mitomycin C (MMC), and Adriamycin (ADM), being cell cycle nonspecific agents, e.g. alkylating agents and anti-tumor antibiotics, and cell cycle specific agents, e.g. antimetabolites. The 6 kinds of chemotherapeutic agents were prepared respectively with 1640 culture solution to form 2-folds of peak plasma concentration (2× PPC) for use. When the solution was used for assay, added 100 μl culture solution which containing equal amount of cells with another 100 μl of the above stock solution, so the concentration of the chemotherapeutic agent was reduced by half, i.e. equal to 1× PPC which were DDP 10.0 mg/L, VDS 1.0 mg/L, 5-Fu 110 mg/L, HCP 5.0 mg/L, MMC 3.0 mg/L, and ADM 10.0 mg/L. Taking 0.2 mg/ml (200 mg/L) verapamil (VPL) (Shanghai Hefeng Pharmaceutical Co. Ltd. China. Verapamil hydrochloride Injection, 5 mg/2 ml) which was equal to 200 folds of the known 1× PPC (0.1 to 1.0 mg/L)[12], added VPL to A549 parental cells, A549 radioresistant cells, and MCF-7 vincristin resistant (MCF7/VCR) cells respectively without chemotherapeutic agents added for the observation of VPL on cell toxicity. Another group was the combined treatment of VPL and chemotherapeutic agent for MCF7/VCR cells.

### Drug sensitiveness experiment of monolayer cell

One 96 well cell culture plate was used, with each group containing 4 wells and the experiment group having 20000 cells per well. The blank well had no cells added, but added with 200 μl culture solution. In the control group, 100 μl culture solution contained cells and another 100 μl culture solution without cell added. As to the ADM blank control group, 100 μl drug containing solution and 100 μl culture solution were added respectively.

### MTT assay methods

Testing cells added with chemotherapeutic drug were cultured for 48 hrs, and then added with 20 μl MTT (5 mg/ml) to every well. After 4 hrs the A value at 490 nm was measured with DG-3022A model enzyme-linked immunosorbent assay instrument (produced by Huadong Electronic Tube Factory, China) and the sensitivity experiment was performed.

### Evaluation of the therapeutic efficacy in MTT experiment

Taking the 1× PPC for the standard in the drug sensitivity experiment, cell survival rate = (A value in the experimental group/A value in the control group) × 100%, and inhibition rate = 1 – cell survival rate. Standard for the evaluation of drug sensitivity was as followed, i.e. Sensitive: 100% > inhibition rate % > 70%; Relatively Sensitive: 70% > inhibition rate % > 20%; Insensitive: 20% > inhibition rate %> 0%.

Use RT-PCR methods for the evaluation of mdrl and MRP multi-drug resistant gene expression in A549 parent cells, A549 radioresistant cells and MCF7/VCR resistant cells****[13,14].

## Results

### Drug sensitivity tests of A549 parent cells and A549 radioresistant cells

At the beginning of the culture, the number of initial cells was the same between the two groups. Two days after being cultured under the same condition without drug added, the A value of parent cells in the control group was 2 times higher than that of the radioresistant cell group, and their A values were 0.635 ± 0.044 and 0.293 ± 0.013 respectively (P < 0.001). It is found that A549 parent cells and A549 radioresistant cells were DDP resistant, but sensitive to VDS, 5-FU, HCP, MMC and ADM, therefore the sensitivity of A549 radioresistant cells to chemotherapeutic drug was about the same as that of their parent cells (Table 1). When treated with VPL but without chemotherapeutic drug added, A549 parent cells had strong toxicity effects with A value 0.017 ± 0.018, but their radioresistant cells had weak toxicity effects with an A value of 0.235 ± 0.026 (P < 0.001), as a result of 98% and 25% inhibition rate to these two cells respectively (Table 1).

**Table 1:** Drug sensitivity tests on A549 parent cells, radioresistant cells and MCF7/VCR resistant cells

No.	Drug	A549 parent cells	A549 radioresistant cells	MCF7/VCR resistant cells	Combine VPL and chemotherapeutic agent for MCF-7 cells
1	DDP	7 Insensitive	0 Insensitive	0 Insensitive	50 Relatively sensitive
2	MMC	93 Sensitive	100 Sensitive	58 Relatively sensitive	83 Sensitive
3	VDS	72 Sensitive	38 Relatively sensitive	0 Insensitive	0 Insensitive
4	ADM	79 Sensitive	97 Sensitive	31 Relatively sensitive	92 Sensitive
5	5-FU	90 Sensitive	100 Sensitive	68 Relatively sensitive	75 Sensitive
6	HCP	91 Sensitive	94 Sensitive	60 Relatively sensitive	83 Sensitive
7	VPL	98 Sensitive	25 Relatively sensitive	80% Sensitive	-

Note: The digit in the results is inhibition rate %, the writing is the explanation of the sensitivity.

### Drug sensitivity experiment on MCF7/VCR cells

MCF7/VCR cells were resistant to DDP and VDS, but were relatively sensitive to MMC, ADM, 5-FU, and HCP. MCF7/VCR cells added with VPL but without chemotherapeutic drug had strong toxicity effects, with an A value of 0.10 ± 0.028 and an inhibition rate of 80% (table 1).

After combined treatment of VPL and chemotherapeutic agents for MCF-7/VCR cells, the relatively sensitivity drugs became sensitive and inhibitive rate of resistant DDP increased 50%, but resistant drug VDS was not reversed (P = 1.00) (table 1).

### Mdr1 and MRP multi-drug resistant gene expression

Using RT-PCR method, *mdr1 *and *MRP *multi-drug resistant gene expressions were assessed in A549 parent cells, A549 radioresistant MTS, re-proliferated cells after irradiation to A549 MTS, and MCF7/VCR resistant cells, while β2-MG serves as the internal reference (Table 2). The results showed that the *Mdr1*/β2-MG in all A549 cells were 0, and the *MRP*/β2-MG gene expressions ranged from 0.3 to 0.7 in all those cells showing variable degrees of reduction after irradiation, but the values of *Mdr1*/β2-MG and *MRP*/β2-MG in MCF7/VCR resistant cells were 35 and 4.36 respectively.

**Table 2:** Semi-quantitative analysis of *mdr1 *and *MRP *in A549 cells, A549 MTS after irradiation, and MCF7/VCR cells

Type of cells	*Mdr1*/β_2_-MG	*MRP*/β_2_-MG
A549 parent cells in single-layer	0	0.76
A549 MTS	0	0.62
A549 MTS, d-9 after 15 Gy ^32^P irradiation [11]	0	0.54
A549 MTS, d-9 after 15 Gy X-ray irradiation [11]	0	0.34
A549 MTS, d-4 after 30 Gy X-ray irradiation[6]	0	0.70
A549 re-proliferated radioresistant cells	0	0.78
MCF7/VCR resistant cells	35	4.36

## Discussion

In this study, re-proliferated cells derived from post-irradiated A549 lung adenocarcinoma MTS were used for an investigation of the disparity of drug sensitivity between the radioresistant cells and their parent cells. It is of great significance for the rational option of chemotherapeutic programs for relapsed cancer after radiotherapy.

The living cell number of A549 MTS became decreased after a medium dose of irradiation, and their mdrl and *MRP *gene expression levels examined by RT-PCR became temporarily reduced, subsequently showed little variation with their parent cells after re-proliferated. In mono-layer culture, A549 re-proliferated radioresistant cell was resistant to DDP, but sensitive to VDS, 5-Fu, HCP, MMC and ADM. The sensitivity to the 6 kinds of chemotherapeutic drugs between the radioresistant cells and their parent cells were almost the same, but the radioresistant cells was resistant to high concentration of VPL, however their parent cells were sensitive to it. The mdrl and *MRP *multi-drug resistant gene expression in MCF7/VCR cells showed a very high level. Moreover, the cells which were resistant or had low sensitivity to a variety of chemotherapeutic agents showed a higher sensitivity to high concentration of VPL than A549 re-proliferated radioresistant cells. VPL had not significant cell toxicity in a concentration of 10 μg/ml. It was found that a high concentration of VPL was needed for the in vitro reversion experiment of drug-resistance. In this article, the concentration of 200× PPC of VPL was used for in vitro experiment. If intra-arterial infusion or chemotherapeutic embolism were used for solid tumor, such a high local concentration can be achieved in the tumor without affecting the general dose and PPC in the body. According to comparative analysis, the reduction of sensitivity of A549 re-proliferated radioresistant cells to VPL was not due to the levels of mdrl and *MRP *multidrug resistant genes. It has been reported in literature that the over-expression of GSH transferase (GST) pi protein in the MCF-7 cells after irradiation leads to an increase of VCR-resistance by 5-times, and the resistance to VP-16 increased by 3-times. In MCF-7 drug resistant sub-line, the selective resistance to some drugs may be related to an increase of the intracellular GST activity [15]. It is generally accepted that the *MRP *drug resistance is indirectly mediated through transporting a compound formed by GS-X and chemotherapeutic agents within the cells. In this article, the *MRP*-resistant gene expression level of A549 re-proliferated radioresistant cell showed no evident elevation, and the parent cells and radioresistant cells were resistant to DDP, which may be due to the increase of GST within the cells [14]. Whether the reduction of VPL sensitivity related to this condition is worthy of further investigation.

VPL is a Ca^2+ ^blocking agent inhibiting the elevation of intracellular calcium and reducing cell death. When the cellular concentration of VPL is high, the drug sensitivity is elevated and consequently the cell death is enhanced. When the inflow of VPL to the radioresistant cells is decreased or the excretion is increased, the drug sensitivity is decreased. Whether the reduced sensitivity of radioresistant cells to VPL is attributable to the formation of protection protein on the surface of the membranous structure awaits further investigations. Apoptosis is involved in Ca^2+ ^flowing into the cytoplasm from endoplasmic reticulum, which can be inhibited by BCL-2. The BCL-2 protein expression is increased in the radioresistant cells [16,17]. Whether the reduction of VPL toxicity is related to the increase of BCL-2 protein is unknown. The pharmacological target of chemotherapeutic drug is DNA, but VPL affects the cell membrane and the calcium passage. It is postulated that, after repairing DNA damage induced by irradiation in A549 pulmonary adenocarcinoma MTS, some changes in membrane proteins may occur. In addition, the MTT test showed that the A value of A549 parent cells was two times higher than their radioresistant cells, which illustrated that the re-proliferate ability of radioresistant cell may be reduced. As a result, the excretion of VPL is increased, leading to the development of VPL resistance. The detailed mechanism is currently unknown.

VPL is generally accepted as a drug resistant reversion agent, but it seems that the radioresistance is different from the multiple drug resistance induced by chemotherapy, and that VPL is probably not an ideal reversion agent for radioresistant cells. Therefore, new strategies need to be developed for the management of the relapse of radioresistant tumors in combination with chemotherapy.

## Competing interests

The authors declare that they have no competing interests.

## Authors' contributions

DS carried out the molecular genetic studies, participated in the cell culture and drafted the manuscript. GS carried out the drug sensitive analysis. GH participated in the tests of internal irradiation with ^32^P. JZ participated in the design of the study and performed the statistical analysis. EL conceived of the study, and participated in its design and coordination. All authors read and approved the final manuscript.
